# Use of Titanium Dioxide Photocatalysis on the Remediation of Model Textile Wastewaters Containing Azo Dyes

**DOI:** 10.3390/molecules161210370

**Published:** 2011-12-14

**Authors:** Enrico Mendes Saggioro, Anabela Sousa Oliveira, Thelma Pavesi, Cátia Gil Maia, Luis Filipe Vieira Ferreira, Josino Costa Moreira

**Affiliations:** 1 Centro de Estudos da Saúde do Trabalhador e Ecologia Humana Escola Nacional de Saúde Pública - Fundação Oswaldo Cruz, Av. Leopoldo Bulhões, 1480, Rio de Janeiro, 21041-210 RJ, Brazil; Email: thelma@fiocruz.br (T.P.); josinocm@fiocruz.br (J.C.M.); 2 CQFM - Centro de Química-Física Molecular and IN - Instituto de Nanociencias e Nanotecnologia, Complexo Interdisciplinar, Instituto Superior Técnico, Universidade Técnica de Lisboa, Av. Rovisco Pais 1049-001, Lisboa, Portugal; Email: LuisFilipeVF@ist.utl.pt (L.F.V.F.); 3 C3i - Centro Interdisciplinar de Investigação e Inovação, Escola Superior de Tecnologia e Gestão, Instituto Politécnico de Portalegre, Lugar da Abadessa Apartado 148 - 7301901 Portalegre, Portugal; Email: catiagmaia@sapo.pt (C.G.M.)

**Keywords:** photocatalytic degradation, semiconductor, photocatalysis, azo dyes, TiO_2_, TiO_2_ reuse

## Abstract

The photocatalytic degradation of two commercial textile azo dyes, namely C.I Reactive Black 5 and C.I Reactive Red 239, has been studied. TiO_2_ P25 Degussa was used as catalyst and photodegradation was carried out in aqueous solution under artificial irradiation with a 125 W mercury vapor lamp. The effects of the amount of TiO_2_ used, UV-light irradiation time, pH of the solution under treatment, initial concentration of the azo dye and addition of different concentrations of hydrogen peroxide were investigated. The effect of the simultaneous photodegradation of the two azo dyes was also investigated and we observed that the degradation rates achieved in mono and bi-component systems were identical. The repeatability of photocatalytic activity of the photocatalyst was also tested. After five cycles of TiO_2_ reuse the rate of colour lost was still 77% of the initial rate. The degradation was followed monitoring the change of azo dye concentration by UV-Vis spectroscopy. Results show that the use of an efficient photocatalyst and the adequate selection of optimal operational parameters may easily lead to a complete decolorization of the aqueous solutions of both azo dyes.

## 1. Introduction

Dyes are an important source of environmental contamination. Textile wastewaters contain usually a considerable amount of unfixed dyes, many of which are azo dyes [1]. It is estimated that fifteen percent of the total World dye production is lost during dyeing process and it is released in textile effluents [2]. The colours produced by minute amounts of dyes accidentally released in water during dying processes are considered to pose serious problems, because they have considerable environmental effects on the water and make them visually unpleasant [3]. Moreover, environmental pollution by organic dyes also sets a severe ecological problem, which is increased by the fact that most of them are often toxic to microorganisms and a have long degradation times in the environment [4].

The number of dyes currently used in textile industry is about 100.000, with over 7 × 10^5^ tons of dye-stuffs being produced annually. Among these dyes, the azo dyes constitute the largest and the most important class of commercial dyes [5,6]. Those dyes, which typically have the chromophoric -N=N- group unit in their molecular structure [7] makes up to 60–70% of all textile dyestuffs produced.

Azo dyes are known to be largely non-biodegradable under aerobic conditions and their stability is proportional to the structural complexity of their molecular structures [7,8]. In order to overcome this problem, azo dyes can be degraded under anaerobic conditions, but causing in this case potentially hazardous and carcinogenic aromatic amines [8,9]. It is well known that azo dye structure when incorporated into the body is split by liver enzymes and intestinal flora into the corresponding aromatic amines, which can cause cancer in humans [7]. Dyes are synthesized to be resistant to fading upon exposure to light, washing and many chemicals [5]. Consequently, traditional wastewater treatment methods such as flocculation, adsorption and biological degradation are usually ineffective [4].

In recent years Advanced Oxidation Processes (AOPs) using titanium dioxide (TiO_2_) have been effectively used to detoxify recalcitrant pollutants present in industrial wastewater [9,10,11,12]. TiO_2 _have singular characteristics that made it an extremely attractive photocatalyst: high photochemical reactivity, high photocatalytic activity, low cost, stability in aquatic systems and low environmental toxicity [13,14]. When a semiconductor such as TiO_2_ absorbs a photon with energy equal to or greater than its band gap width (3.2 eV), an electron may be promoted from the valence band to the conduction band (e^−^_cb_) leaving behind an electron vacancy in the valence band (h^+^_vb_) [15]. The holes at the TiO_2_ valence band, having an oxidation potential of +2.6 V can oxidize water or hydroxide to produce hydroxyl radicals. The hydroxyl radical is a powerful oxidizing agent and enables a non- specific attack on organic compounds; under favorable conditions the final photoproducts are H_2_O, CO_2_ and inorganic anions. The general detailed mechanism of dye degradation upon irradiation is described by Equations 1–6 [4,15]:

Dye + *hν* → Dye* (1)

Dye* + TiO_2_→ Dye•^+ ^+TiO_2_(e) (2)

TiO_2_ (e) + O_2_→ TiO_2_+ O_2_^•− ^ (3)

O_2_^•−^ + TiO_2_ (e) + 2H^+^ → H_2_O_2_ (4)

H_2_O_2_+ TiO_2_ (e) → ^•^OH + OH^−^ (5)

Dye•^+ ^+ O_2_ (or O_2_^•−^ or ^•^OH)→ peroxylated or hydroxylated intermediates

→ degraded or mineralized products (6)

Nowadays it is well known that TiO_2_ is one of the most suitable semiconductors for photocatalysis and has been applied into various photocatalytic reactions [16,17,18,19,20,21,22,23,24,25,26]. The aim of the present work was to investigate the influence of various parameters on the photocatalytic degradation of two textile azo dyes, namely C.I Reactive Black 5 and C.I Reactive Red 239, by UV-light irradiation in the presence of TiO_2_. In this paper, we will also examine the effects of the amount of TiO_2_ used, pH, initial concentration of the dyes, the use of an electron acceptor such as hydrogen peroxide and test the efficiency of TiO_2 _recycling and reuse. 

## 2. Results and Discussion

### 2.1. Effect of TiO_2_ Photocatalyst Concentration

**Figure 1:**
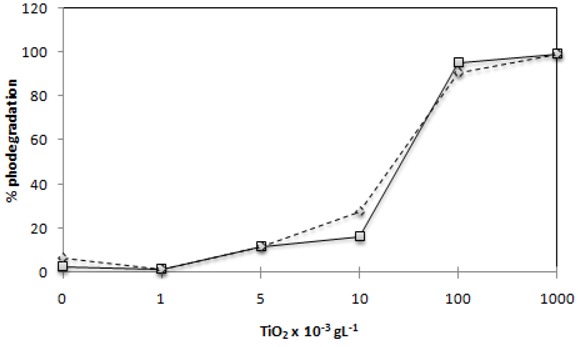
Effect of TiO_2_ amount on the complete degradation of 30 mg·L^−1^.I. Reactive Red 239(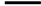) and C.I Reactive Black 5 (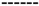) dyes in 120 min of irradiation with2,60 mW/cm^2^ of irradiation power by a 125 W mercury lamp.

The photocatalytic degradation of the two azo dyes (30 mg·L^−1^) with different TiO_2_ amounts was studied according to the described below in Section 3.3 and the obtained results are shown in Figure 1. The efficiency of photocatalytic degradation of both dyes clearly increased with the increase of the amount of photocatalyst. The concentration of 1 g·L^−1^ of TiO_2_ degraded around 90% of both dyes in about 45 min and 99% of degradation was attained after 120 min. However, the concentration of 0.1 g·L^−1^ of TiO_2_ in the end of 120 min of irradiation yielded a similar degradation to that of the highest concentration of TiO_2 _used. This can be rationalized in terms of availability of active sites on the TiO_2_ surface and of the light penetration of photoactivating light into the dye-TiO_2_ suspension; in fact in the solutions of both azo dyes, containing 1 g·L^−1^ of TiO_2_ in suspension the light penetration depth is considerably smaller than in those containing only 0.1 g·L^−1^ of TiO_2_ and so the effect of the increase on the amount of photocatalyst becomes reduced. For the two azo dyes studied in this work the treatment with TiO_2_ under artificial irradiation of a 125 W mercury vapor lamp was an extremely efficient photodegradation method, since after 2 hours all dyes showed substantial colour losses. 

The photodegradation is mostly promoted by titanium dioxide since we observed that direct photolysis, in the two hours irradiation period, is only responsible for the degradation of 0 to 6% of both of these azo dyes. Therefore, under irradiation, only the molecules adsorbed on the surface of TiO_2_ can be degraded. When the amount of catalyst used on the photocatalytic degradation is very high the turbidity of the suspension strongly inhibits further light penetration in the photoreactor [26,27]. This limit on TiO_2_ amount to be used depends on the geometry and working conditions of the photoreactor which should enable that all the photocatalyst particles present on the entire exposed surface are fully illuminated [28]. In this way, for each system to be remediated thought this method, the optimum amount of TiO_2_ has to be determined in order to avoid the use of unnecessary catalyst in excess and also to ensure the total absorption of the irradiating photons in order to achieve an efficient photoremediation.

### 2.2. Effect of UV-Irradiation Time

**Figure 2:**
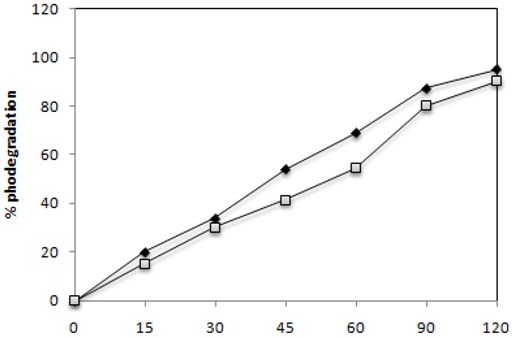
Effect of UV-Irradiation time on the degradation of 30 mg·L^−1^of the azo dyesC.I. Reactive Red 239 (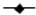) and C.I Reactive Black 5 (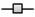) with 0.1 g·L^−1^ of TiO_2_ and 2.60 mW/cm^2^ of irradiation power by a 125 W mercury lamp.

**Figure 3:**
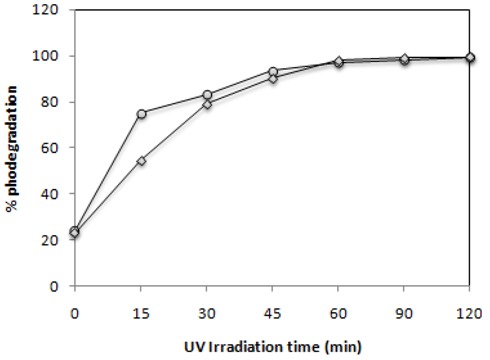
Effect of UV-Irradiation time on the degradation of 30 mg·L^−1^of the azo dyesC.I. Reactive Red 239 (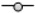) and C.I Reactive Black 5 (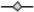) with 1 g·L^−1^ of TiO_2_ and 2.60 mW/cm^2^ of irradiation power by a 125 W mercury lamp.

Figure 2 and Figure 3 show the effect of UV-light irradiation time on the photocatalytic degradation of C.I. Reactive Black 5 and C.I. Reactive Red 239 with the two most efficient added amounts of TiO_2_, 0.1 g·L^−1^ and 1 g·L^−1^, respectively. In the presence of 1 g·L^−1^ of TiO_2_ and UV-light, 75 and 79% of the two azo dyes were degraded, respectively, after an irradiation time of 30 min. It is also evident that for both of them the percentage of decolorization and photodegradation increases with increasing irradiation time. As expected the concentration of 1 g·L^−1^ of TiO_2_ promoted the highest percentage of degradation for the two dyes. This concentration degraded 90 to 93% in 45 min of both azo dyes. The rate of degradation became slower after 45 minutes. By the end of two hours up to 90 to 95% of the azo dyes were degraded by 0.1 g·L^−1^ of catalyst. However, 1 g·L^−1^ degraded up to 93 to 99% of dyes for the longest irradiation times.

The slow kinetics of azo dyes degradation after a long time of irradiation arises from the difficulty in converting the N atoms of the dyes into oxidized nitrogen compounds, since aliphatic chain interaction with hydroxyl radicals is small and these radicals are short lived [29]. The quick lost of colour of both azo dyes solutions was associated with cleavage of the azo linkage in dyes molecules. The nitrogen to nitrogen double bonds (-N=N-) are characteristic of azo dyes molecules and theirs colours are determined by azo bonds. Azo bonds are the most active bonds in azo dye molecules and can be easily oxidized either by positive holes or hydroxyl radicals [30,31].

**Figure 4:**
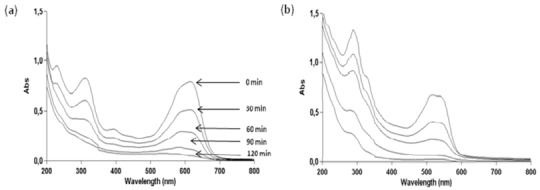
Photocatalytic degradation under 125 W mercury-vapor lamp irradiation with 0.1 g·L^−1^ of TiO_2_ followed by UV-Vis spectrophotometry from 200 to 900 nm for 30 mg·L^−1^ of (a) C.I Reactive Black 5 and (b) C.I Reactive Red 239.

The cleavage of double bonds leads to the decolourization of the dye. The UV-Vis spectra measured during photodegradation of the two commercial azo dyes are shown in Figure 4. Before irradiation, C.I Reactive Black 5 exhibits peaks at 615, 397, 312 and 231 nm and C.I. Reactive Red 239 exhibits peaks at 540, 328, 289 and 215 nm. The visible spectrum peaks are due to chromophoric group absorptions, whereas the bands observed in the UV region can be assigned to the aromatic rings present in both azo dye structures, as can be observe in Figure 10 [32,33]. Figure 4 shows for both dyes the fast decolorization observed and also a significant degradation of their aromatic structures.

### 2.3. Effect of pH

**Figure 5:**
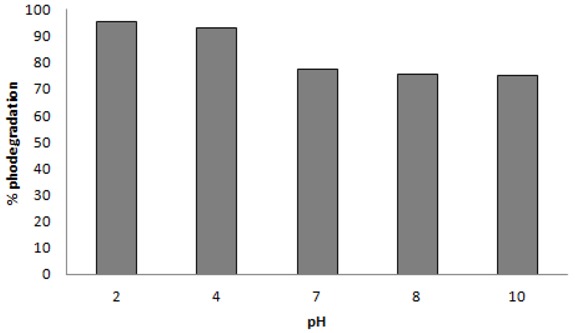
TiO_2_ (0.1 g·L^−1^) photodegradation efficiency of 30 mgL^−1 ^C.I Reactive Black 5 at different pH values.

pH is an important parameter for reactions taking place on the surface of a particulate, as is the case of TiO_2_ photocatalysis. pH variation can in fact influence the adsorption of dye molecules onto the TiO_2_ surfaces [34]. The effect of solution pH was studied in the range of 2 to 10 for the optimized catalyst amounts (*i.e.*, 0.1 gL^−1^) and UV-irradiation times (up to 120 min) for the azo dyes under study. Figure 5 shows the variation on the efficiency photocatalytic degradation of C.I Reactive Black 5 at different pH values. Photodegradation was higher in acidic media (pH 2 to 4), with degradation rates of 95 and 93%, respectively. Up to a pH value of 7, the dye degradation efficiency decreased to 77%. Above pH 7, the degradation continued to decrease to about 70% at pH 10.

**Figure 6:**
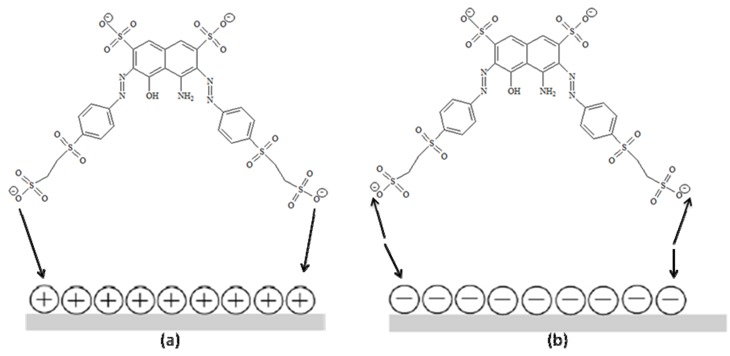
Schematic interaction model of C.I Reactive Black 5 and TiO_2_: (a) acid sites and (b) basic sites.

This catalyst behavior can be explained by TiO_2_ surface charge density. The point of zero charge (pzc) of the TiO_2_ (Degussa P25) is at pH 6.8. In acid media (pH ≤ 6.8) the TiO_2_ surface is positively charged, whereas under alkaline conditions (pH ≥ 6.8) it is negatively charged [25,35]. Considering the structure of C.I Reactive Black 5 (Figure 10a), a positive charge excess in the TiO_2_ surface promotes a strong interaction with SO_3_^−^ groups of the dye (Figure 6a). A negative charge excess promotes the repulsion of the dye by the titanium surface, diminishing the catalytic activity of this semiconductor (Figure 6b). These results suggest that the influence of the initial pH of the solution on photocatalysis kinetics is due to the amount of the dye adsorbed on TiO_2_ [23,34]. This hypothesis agrees with a reaction occurring at TiO_2_ surface and not in the solution, close to the surface.

### 2.4. Recycling of TiO_2_

**Figure 7:**
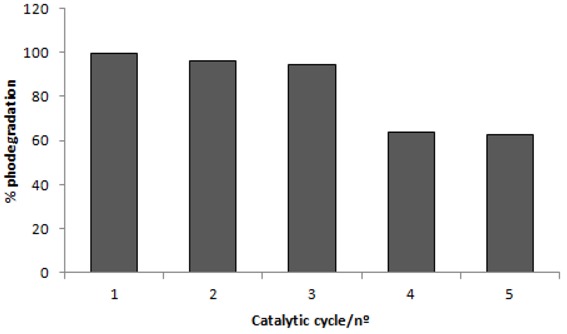
Catalytic yields of 0.1 g·L^−1^of TiO_2_ as a function of its reuse of 30 mg·L^−1^of C.I. Reactive Red 239 photodegradation.

One of today’s main industrial wastewater treatment strategies is focused on the development of green technologies and management practices for environmental benefit. Then, TiO_2_ recycling can be foreseen as a good practice for sustainable wastewater treatment. Consequently, it is necessary to demonstrate whether, after a photocatalytic treatment, the catalyst can be reused. The TiO_2_ catalyst was used and recycled for consecutive reuse on the C.I. Reactive Red 239 degradation; the process was repeated up to five times. The TiO_2_ recycling studies were performed with 0.1 g·L^−1^ of the catalyst and the efficiency of the photodegradation process was evaluated and compared between the reuse cycles, as shown in Figure 7.

These studies reveled that TiO_2_ demonstrated good stability after recovery and that catalyst reuse is effective. The first cycle degraded 99% of the dye after 60 minutes of irradiation. Subsequently, the second and third cycle degraded 96 and 94% the dye, respectively. After these cycles, the efficiency markedly decreased, as demonstrated in the fourth and fifth cycles, where the rates of degradation felt to 63 and 62% respectively. However, the rate of degradation is still significant after five times of TiO_2 _reuse. Agglomeration and sedimentation of the dye around TiO_2_ particles after each cycle of photocatalytic degradation is a possible cause of the observed decrease on the degradation rate, because each time the photocatalyst is reused new parts of the catalyst surface become unavailable for dye adsorption and thus photon absorption, reducing the efficiency of the catalytic reaction.

An alternative to regenerate TiO_2_ after each usage is to apply H_2_O_2_ under UV irradiation [36]. Wang *et al.* [37] used TiO_2 _modified by photocatalytic degradation through six successive batches and obtained successively similar rates of degradation, demonstrating that the H_2_O_2_ coated TiO_2_ have an enhanced photochemical stability for reuse. Xie and Yuan [38] utilized a pure recycled TiO_2_ in the degradation of an organic dye and observed that the removal ratio kept above 90% after three times or reuse, and above 77% after five times of reuse. This ability of titanium dioxide to be reused goes towards green chemistry key principles. 

### 2.5. Effect of Dye Concentration

**Figure 8:**
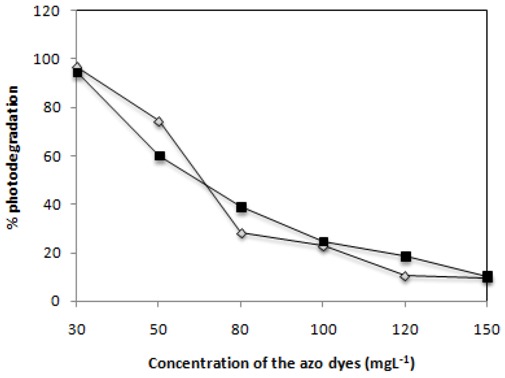
Effect of the initial concentration of C.I. Reactive Red 239 (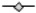) and C.I Reactive Black 5 (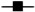) dyes on the efficiency attained after 2 h of irradiation with 0.1 g·L^−1^ of TiO_2_ and 2,60 mW/cm^2^ of irradiation power by a 125 W mercury lamp.

The effect of the initial concentration of C.I Reactive Black 5 and C.I. Reactive Red 239 on the decomposition of the dye under the 125 W mercury lamp reactor was determined. The obtained results are presented in Figure 8. The results indicate that the decomposition rate of both dyes strongly depends on the initial dye concentration. The efficiency of photodegradation of both dyes decreased with increase of the initial dye concentration.

C.I Reactive Black 5 and C.I. Reactive Red 239 with 30 mg·L^−1^ of photocatalyst show rates of degradation of 95 and 97%, respectively. On increasing the concentration of the dye until 150 mg·L^−1^ the photodegradation became very slow, presenting a degradation of only 9% for C.I. Reactive Red 239 and 10% for C.I Reactive Black 5. As the initial concentration of the dye increased, more dye molecules were adsorbed on the surface on the catalyst, consequently the generation of hydroxyl radicals was reduced since the active sites were occupied by dyes [24,39,40]. An increase of the initial dye concentration results in an increase of the amount of dye adsorbed on the catalyst surface, affecting the catalytic activity of the photocatalyst [41,42]. Moreover the reduction of the light path length as the concentration and deepness of the colour of the solution rises also cannot be neglected.

### 2.6. Effect of H_2_O_2_

**Figure 9:**
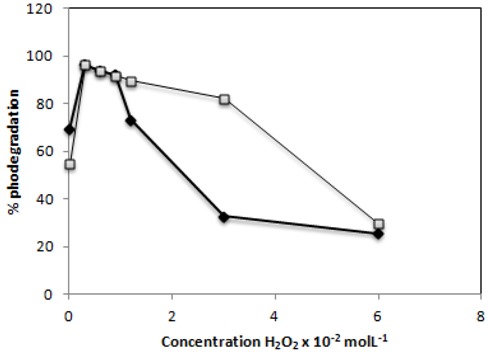
Effect of the presence of hydrogen peroxideon the photodegradation of C.I. Reactive Red 239 (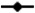) and C.I Reactive Black 5 (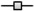) dyes after 60 min of irradiationwith 0.1g−L^−1^ of TiO_2_ and 2,60 mW/cm^2^ of irradiation power by a 125 W mercury lamp.

Since hydroxyl radicals appear to play an important role in the photocatalytic degradation, electron acceptors such as hydrogen peroxide were added to the dye solution. Hydrogen peroxide has been found to enhance the degradation of compounds due to a more efficient generation of hydroxyl radical and inhibition of electron/hole pair recombination. The degradation rates for the C.I Reactive Black 5 and C.I. Reactive Red 239 in presence of UV/TiO_2_/H_2_O_2_ in order to find the optimal H_2_O_2 _concentration to be used are shown in Figure 9. It was observed that the added hydrogen peroxide had a beneficial effect on the degradation of both azo dyes. The maximum reaction rate was observed with 0.3 × 10^−2^ mol·L^−1^ of H_2_O_2_ for both dyes (achieving a 96% degradation rate in 60 minutes). The two H_2_O_2_ concentrations below the latter (0.6 × 10^−2^ and 0.9 × 10^−2^ mol·L^−1^) produced satisfactory and similar photocatalytic degradation rates (93 and 91%) for both dyes. At higher concentrations of H_2_O_2_ the degradation efficiency decreased significantly for C.I. Reactive Red 239, with only 25% of degradation being obtained with the highest concentration of H_2_O_2_ used. For C.I Reactive Black 5 respectively 89 and 82% of degradation were achieved with 1.2 × 10^−2^ mol·L^−1^ and 3 × 10^−2^ mol·L^−1 ^of H_2_O_2_, but the highest concentration of H_2_O_2_ used degraded just 29%.

The electron/hole recombination is a problem in photocatalytic degradation in presence of the TiO_2_, and then one strategy to inhibit electron-hole pair recombination is to add other electron acceptors to the reaction [43]. When present at a low concentration, hydrogen peroxide enhanced the degradation rate, a fact that could be attributed to a suitable trapping of electrons by hydrogen peroxide thereby preventing the recombination of e^−^ and h^+^ pairs and thus increasing the chances of formation of hydroxyl radicals on the surface of the catalyst [44,45]. However, when the concentration of H_2_O_2_ increases, the electron acceptor reacts with hydroxyl radicals, and acts as scavenger of the photoproduced holes. In addition, H_2_O_2_ can modify the TiO_2_ surface. This fact probably decreases its photocatalytic efficiency [46].

### 2.7. Effect of the Mixture of the Two Azo Dyes

**Figure 10:**
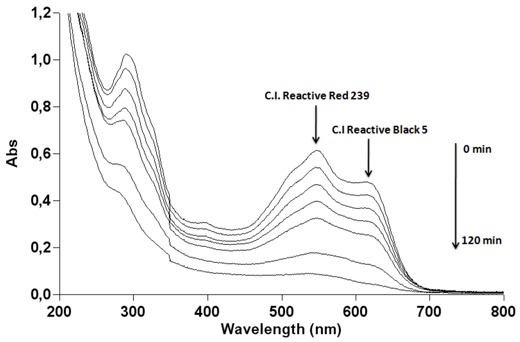
Photocatalytic degradation under 125 W mercury-vapor lamp irradiation with 0.1 g·L^−1^ of TiO_2_ followed by UV-Vis spectrophotometry from 200 to 900 nm for a 30 mg·L^−1^ mixture of C.I Reactive Black 5 and C.I Reactive Red 239.

**Figure 11:**
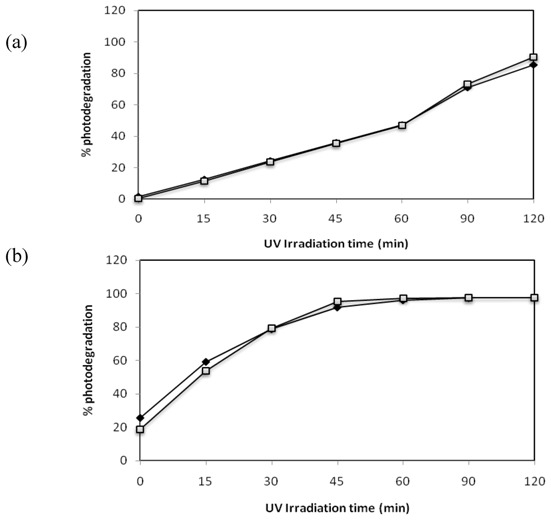
Effect of UV-Irradiation time on the degradation of a 30 mg·L^−1^mixture of the azo dyesC.I. Reactive Red 239 (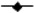) and C.I Reactive Black 5 (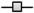) with (a) 0.1 g·L^−1^ and (b) 1 g·L^−1^ of TiO_2_ and 2.60 mW/cm^2^ of irradiation power by a 125 W mercury lamp.

Since in real effluents from dye industries the presence of several dyes in the waters is frequent we decided to test the effect of the simultaneous presence in solution of both azo dyes under study in this work. The obtained results are present below in Figure 10 and Figure 11.

The photocatalytic degradation of the mixture of the two azo dyes was very satisfactory. C.I Reactive Red 239 was degraded 96% with 1 g·L^−1^ of TiO_2 _after 120 min of irradiation and when in the presence of C.I Reactive Black 5 in solution; the same results (97% degradation) have been observed when the dye in solution was irradiated alone in the same conditions. When using 0.1 g·L^−1^ of TiO_2 _the degradation observed is also similar after 120 min of irradiation either for monocomponent solutions or bi-component solutions. C.I Reactive Black 5 achieved a similar degradation for both concentrations of TiO_2_ in monocomponent solutions and bi-component solutions. The simultaneous presence of the two azo dyes in solution did not disturb the photocatalytic degradation of each of them. The degradation rates achieved in mono and bi-component system were identical. These results obtained for azo dyes suggest that TiO_2_ can be efficiently used in complex systems containing more than one organic molecule to be degraded, because TiO_2_is a powerful oxidizing agent and enables a non- specific attack to organic compounds, as Bergamini *et al.* demonstrated [22]. 

## 3. Experimental

### 3.1. Reagents and Materials

Marine Remazol RGB 150% gran (C.I Reactive Black 5, M.W. = 981.82 g/mol), Ultra Red Remazol gran (C.I. Reactive Red 239, M.W. = 1085.84 g/mol) textile dyes were obtained from DyStar (Brazil). The chemical structure of the dyes is shown in Figure 10. These compounds were used as received from the supplier without any further purification. The photocatalyst used was titanium dioxide, Degussa P25, which consists of 75% anatase and 25% rutile with a specific BET - surface area of 50 m^2^·g^−1^ and a primary particle size of 20 nm. The other chemicals used in this study such NaOH, HCl and H_2_O_2_ were obtained from Merck. Water used to prepare dye solutions was MiliQwater.

**Figure 12:**
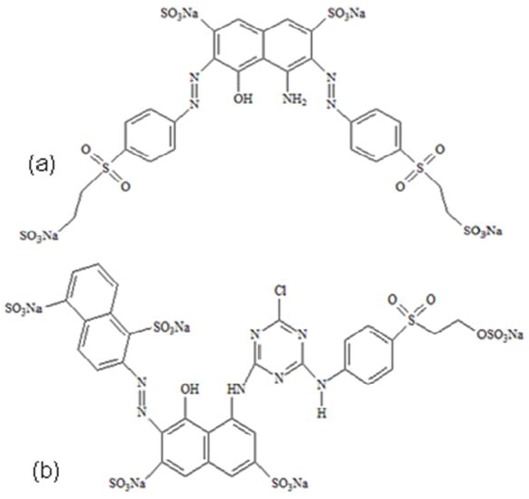
Chemical structure of commercial azo dyes: (a) Marine Remazol RGB 150% gran (C.I Reactive Black 5). (b) Ultra Red Remazol gran (C.I. Reactive Red 239).

### 3.2. Experiments Procedure

Fresh solutions of the dyes were always prepared just before use and diluted according to the requirements of the experiments. The photodegradation cell was feed with 100 mL of the solution with stirring. Before irradiation, each sample was kept in the dark for 40 min. During irradiation, continuous stirring was maintained to keep the suspension homogenous. Samples (ca. 3 mL) were withdrawn at specific times (0, 15, 30, 45, 60, 90 and 120 min) for UV-Vis analysis and centrifuged for 10 minutes at 1,000 rpm. The concentration of the azo dyes in each sample was evaluated spectrophotometrically measuring the UV-Visible absorption at the maximum absorption wavelength of each dye (C.I Reactive Black 5; *λ*_max_ = 615 nm; C.I. Reactive Red 239; *λ*_max_ = 540 nm) and comparing it with the dye concentration calibration curve of each of the azo dyes used.

The samples were irradiated with a 125 Watts mercury vapour lamp (HQL 125 watts, from Osram). In this type of commercial lamps, the filament is protected by a glass bulb that cuts all UV-A and UV-B radiation. The glass bulb presents a white colour due to the internal phosphor coating that improves the radiation of the lamp on the visible region. This type of lamps with glass bulb is appropriated to selectively illuminate and exclusively excite TiO_2_ band gap, avoiding direct photolysis of the dye molecules that could be simultaneously promoted if all the lamp emission profile was available. The photoreactor is composed of an elliptical cover that supports the irradiation source described above and of a base containing a magnetic stirrer, where the samples to be irradiated are placed in 100 mL beakers. The light emitted from the mercury lamp was measured at 366 nm (the wavelength of TiO_2_ bandgap [47]) with the help of a Cole Parmer radiometer (series 9811-50) placed above the beaker with the sample to be irradiated. All samples were illuminated with an irradiation power of 2.6 ± 0.2 mW/cm^2^.

### 3.3. Effect of TiO_2_ Photocatalyst Concentration

A 30 mg·L^−1^ dye solution of each azo dye (100 mL) containing different concentrations of TiO_2_ (0, 1 × 10^−3^, 5 × 10^−3^, 1 × 10^−2^, 1 × 10^−1^ and 1 g·L^−1^) were irradiated in the photoreactor described above for 120 min.

### 3.4. Effect of pH

A solution of 30 mg·L^−1^C.I Reactive Black 5 and 0.1 g·L^−1^ of TiO_2_ (100 mL) was used to degrade the azo dye solution at different pH values, which were adjusted by addition of HCl and NaOH. Irradiation in the photoreactor described above was carried out for 60 min.

### 3.5. Recycling of TiO_2_

The recycling of the photocatalyst was performed as follows: after a first photodegradation cycle of a 30 mg·L^−1^ solution of C.I. Reactive Red 239 (100 mL) using 0.1 g·L^−1^ of TiO_2 _and 60 min irradiation, the treated solution of the dye was centrifuged with a rotation of 4,000 rpm for 15 minutes to remove TiO_2_. The liquid phase was filtered by a vacuum system with a Millipore membrane (0.45 μm) and the solid phase containing the photocatalyst was carefully separated for reuse. After allowing it to dry in a desiccator with silica gel, the separated catalyst was added again to a new identical batch of C.I. Reactive Red 239 to be remediated. The process was repeated 5 times. 

### 3.6. Effect of Dye Concentration

Different concentrations of each azo dye solutions (30, 50, 80, 100, 120 and 150 mg·L^−1^, 100 mL) containing 0.1 g·L^−1^ of TiO_2_ were illuminated in the photoreactor for 120 min.

### 3.7. Effect of H_2_O_2_

Thirty mg·L^−1^ dye solution of each azo dye (100 mL) containing 0.1 g·L^−1^ of TiO_2_ and different concentrations of hydrogen peroxide (0, 3 × 10^−3^, 6 × 10^−3^, 9 × 10^−3^, 1.2 × 10^−2^, 3 × 10^−2^ and 6 × 10^−2^ mol·L^−1^) were illuminated in the photoreactor for 60 min.

### 3.8. Effect of the Mixture of the Two Azo Dyes

Thirty mg·L^−1^ dye solutions of each azo dye under study (50 mL) were added in a beaker in order to have a 100 mL mixed solution of the two azo dyes. The photodegradation of the mixture of the two azo dyes was tested with two different concentrations of TiO_2_ (1 g·L^−1^ and 0.1 g·L^−1^) and were irradiated in the photoreactor described above for 120 min.

### 3.9. Equipment

A double beam UV-Visible spectrophotometer (Shimadzu UV-1601PC) was used for the spectrophotometric determination of azo dyes absorption spectra from 200–900 nm. Spectra of the dyes in water were recorded with the help of 1 cm quartz cuvettes.

## 4. Conclusions

The photocatalytic degradation of two commercial azo dyes mediated by TiO_2_ was successfully achieved. Results indicated that the photocatalytic degradation of two dyes in water with powdered TiO_2_ depended on the concentration of dye, amount of photocatalyst used, UV-irradiation time, solution pH and concentration of added hydrogen peroxide. It was found that the optimal amount of catalyst to be used was 0.1 g L^−1^. Concerning the initial dye concentration, it has been observed that the increase in the initial dye concentration leaded to a decrease in photodegradation. The photodegradation is favored in acidic solution. The optimal H_2_O_2_ concentration to be added was found to be 3 × 10^−3^ mol·L^−1^. The recycling of TiO_2_ can be performed with the photocatalyst being able to be adequately used in other reactions. The TiO_2_ has the same photocatalytic activity in reactions with monocomponent solutions and bi-component solutions. 
